# Development of an Antigen-DNAzyme Based Probe for a Direct Antibody-Antigen Assay Using the Intrinsic DNAzyme Activity of a Daunomycin Aptamer

**DOI:** 10.3390/s140100346

**Published:** 2013-12-27

**Authors:** Noorsharmimi Omar, Qiuting Loh, Gee Jun Tye, Yee Siew Choong, Rahmah Noordin, Jörn Glökler, Theam Soon Lim

**Affiliations:** 1 Institute for Research in Molecular Medicine, Universiti Sains Malaysia, Minden 11800, Penang, Malaysia; E-Mails: mimiez_m5@yahoo.com.my (N.O.); qiutingloh@gmail.com (Q.L.); geejun@usm.my (G.J.T.); yeesiew@usm.my (Y.S.C.); rahmah@usm.my (R.N.); 2 Department of Molecular Biotechnology and Functional Genomics, Technical University of Applied Sciences Wildau, Bahnhofstr. 1, Wildau 15745, Germany; E-Mail: gloekler@th-wildau.de

**Keywords:** G-quadruplex, DNAzyme, gold nanoparticles, antibody

## Abstract

G-Quadruplex (G-4) structures are formed when G-rich DNA sequences fold into intra- or intermolecular four-stranded structures in the presence of metal ions. G-4-hemin complexes are often effective peroxidase-mimicking DNAzymes that are applied in many detection systems. This work reports the application of a G-rich daunomycin-specific aptamer for the development of an antibody-antigen detection assay. We investigated the ability of the daunomycin aptamer to efficiently catalyze the hemin-dependent peroxidase activity independent of daunomycin. A reporter probe consisting of biotinylated antigen and daunomycin aptamer coupled to streptavidin gold nanoparticles was successfully used to generate a colorimetric readout. In conclusion, the daunomycin aptamer can function as a robust alternative DNAzyme for the development of colorimetric assays.

## 1. Introduction

Aptamers have been applied for various applications, including affinity purification [1], drug discovery [2], high-throughput screening [3], therapeutics [4,5] and diagnostics [6]. Aptamers are selected via an in vitro process called Systematic Evolution of Ligands by Exponential Enrichment (SELEX), an iterative process of selection/isolation and amplification from a large combinatorial library of oligonucleotides [7]. Daunomycin is an anthracycline antibiotic commonly used as a cancer chemotherapeutic agent [8]. Daunomycin is known to prefer G-C-rich DNA by intercalation with the pyridine and pyrimidine nucleobases [9]. The daunomycin aptamer was predicted to form a G-4 which is likely the basis of its binding conformation [10]. Previous structural analysis of the hemin G-4 structure shows that the hemin is positioned at the planar ends of the G-4 [11–13]. A recent study on the crystal structure of the G-4 complex with daunomycin shows that the interaction between daunomycin to the G-4 occurs by van der Waals interaction with a substantial π - π stacking effect. It shows that 5′-guanine adopts an unusual *syn* glycosyl linkage instead and no ligand-quadruplex groove insertion interaction exists [12]. Both structures show that each hemin and daunomycin is stacked in a similar fashion, allowing these molecules to be sandwiched together between the G-4 planes.

Nucleic acid chains with repetitive G-rich motifs can fold into a G-quadruplex through hydrogen bonds. It is stabilized by the presence of cations and interacts with hemin (an iron containing porphyrin) forming a G-quadruplex-hemin complex mimicking the horseradish peroxidase enzymatic activities. Alternatively, a specific nucleic acid sequence may develop a defined structure that can react as a catalyst called DNAzyme. As DNAzymes gain momentum in applications of various fields, many attempts have been made to utilize the application of known DNAzymes [14] to detect mainly nucleic acids and metal ions. Detection of proteins by DNAzymes is mostly combined with an antigen-specific aptamer but rarely with an antibody [15]. Many immunosensor designs are also based on DNAzymes conjugated onto solid phases like magnetic nanoparticles (MNPs) or gold nanoparticles (AuNPs) [16]. Many have reported DNAzymes as the reporter system replacing the natural enzymes used in conventional immunoassays [17,18]. Conventional ELISA methods require enzymes like horseradish peroxidase to be conjugated to an antibody or antigen [19]. For many DNAzyme applications whereby biotinylated oligonucleotides are easily synthesized, the highly specific streptavidin-biotin interaction can be used to substitute the conjugation process.

**Figure 1.**
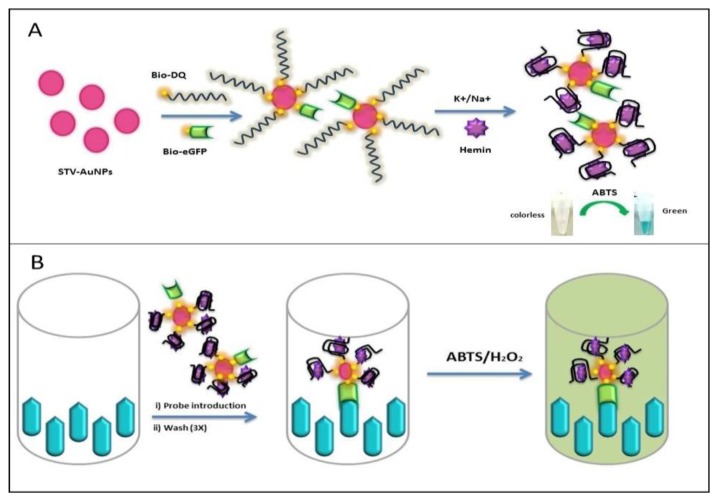
Schematic diagram of STV-AuNPs and Ag-Ab/DNAzyme conjugation as probe (**A**) for immunoassay system (**B**).

Here, we apply the generation of an antigen-DNAzyme based probe for detection. The probe takes advantage of the specificity that biotinylated antigen and biotinylated oligos have towards multivalent streptavidin on nanoparticles for the generation of an antigen-DNAzyme complex. The use of streptavidin nanoparticles in the proposed reporter system allows for one-pot synthesis of the reporter system for rapid assays (Figure 1). This reporter system allows for the application to direct and competitive assays which can be beneficial for the detection of small haptens such as hormones or drug molecules. Therefore the proposed probe can function as an alternative reporter system for general immunoassay applications.

## 2. Experimental Section

### 2.1. Materials

Daunorubicin hydrochloride (daunomycin) and hemin were purchased from Sigma Aldrich (St. Louis, MO, USA) and subsequently dissolved to 5 mM in dimethyl sulfoxide (Merck, Darmstadt, Germany) as stock solution. The streptavidin-gold nanoparticles (STV-AuNP) at 40 nm diameter, 7.15 × 10^10^ nanoparticles/mL was bought from Sigma Aldrich. ABTS was prepared by dissolving 10 μL of 100% H_2_O_2_ in sodium citrate buffer (Merck). 96-well plate for absorbance reading was purchased from Corning (Corning, NY, USA).

### 2.2. Oligonucleotides

The G-rich oligonucleotides sequences, control hemin G4 oligonucleotide d(G3AATTCGAGCT CG2TACCTG3TAG3CG3TTG3AAA) and daunomycin G4 oligonucleotide d(G3AATTCGAGCT CG2TACCATCTGTGTAAG4TAAG4TG5TG3TACGTCTAG) were synthesized by Integrated DNA Technologies (Coralville, IA, USA). All oligonucleotides were synthesized with the addition of biotin at the 5′-end. The oligonucleotides stock solutions (10 μM) were prepared in Millipore Milli-Q water and kept at −20 °C.

### 2.3. Preparation of G-Quadruplex Complexes

To form the secondary G-quadruplex structure, 10 μL of 10 μM aptamers were heated at 88 °C for 10 min to dissociate intermolecular interactions and slowly cooled to room temperature (RT) for 1 h. 10 μL of 2× HEPES buffer (50 mM HEPES, 40 mM KCl, 400 mM NaCl, 0.1% Triton-X, 2% DMSO, pH 7.2) was added together and incubated for 1 h to form G-quadruplex structures [20]. Next, 5 μL hemin (5 mM) was added to the reaction mixture and incubated at RT for 1 h giving way to the complexation of hemin with the G-quadruplex.

### 2.4. UV-Visible Scanning Analysis of DNAzyme

G-quadruplex complexes after addition of 100 μL ABTS were left to react for 10 min and further diluted to 2 mL volume mix with 0.5 X HEPES buffer for spectral analysis. The scanning was done using a NIR 3600 UV-Visible spectrophotometer (Shimadzu, Kyoto, Japan, 300–700 nm).

### 2.5. Absorbance Analysis of DNAzyme

Two hundred μL of ABTS-H_2_O_2_ solution was added into the samples and photometric analysis was performed at the absorbance wavelength of 405 nm. A Multiskan spectrophotometer (Thermo Fisher Scientific, Waltham, MA, USA) was used for the photometric endpoint measurements of the ABTS-H_2_O_2_ reaction.

### 2.6. Expression of Biotinylated Green Fluorescent Protein (eGFP) and scFv Antibody

Single chain fragment variable (scFv) antibody against GFP (clone G11) was cloned and expressed in BL21 *E. coli* with C-terminus histidine tag (His6–Tag). Biotinylated green fluorescent protein (Bio-eGFP) clone containing the Avi-tag was cloned and expressed in BL21 *E. coli* containing a helper plasmid expressing biotin ligase. 500 mL of 2YT media was supplemented with 100 μg/mL ampicillin, 34 μg/mL chloramphenicol and 0.2% glucose. The cells were grown at 37 °C, at 200 rpm to an OD600 of 0.8 and induced with 1 mM IPTG and further incubated at 30 °C until overnight. The bacteria cells were pelleted through centrifugation followed by sonication and further purified with Ni-NTA column. Both purified protein fractions were run on 12% SDS-PAGE gel. The biotinylated eGFP was additionally assayed with the streptavidin-HRP to confirm successful biotinylation using ELISA.

### 2.7. Generation of STV-AuNPs-antigen (Ag)–DNAzyme Probe

Thirty μL of STV-AuNPs solution (6 μg/mL STV conjugated) was mixed with bio-eGFP and bio-daunomycin aptamer (bio-DQ) with the ratio of 1:100 by stirring, allowing two different biotinylated molecules to bind STV-AuNPs [21]. All the unbound molecules were washed away with PBS containing 0.1% Tween-20 and the complex was separated by a magnet. Three hundred μL PTM (2% skimmed milk in PBS) solution was added to block excess reactivity of STV-AuNPs for 1 h. The gold complex was washed again and the DNAzyme was formed with a 10 fold higher amount of cations (100 μL of 2× HEPES buffer) and hemin (50 mM). The final probe was diluted to 1 mL of 0.5× HEPES buffer and kept at 4 °C until use.

### 2.8. Antigen-DNAzyme Probe Immunoassay System

Microtiter plates were coated with 20 μg anti-eGFP (scFv G11) in PBS buffer (pH 7.4) for 3 h at room temperature and blocked with PTM solution for 1 h. Then, plates were incubated with the 200 μL of the probe for 1h. Wells were washed three times with PBS containing 0.01% Tween-20. Finally, the wells were developed with ABTS solution (200 μL/well) for 30 min at 37 °C with mild shaking.

## 3. Results and Discussion

### 3.1. Characterization of the Hemin and Daunomycin Quadruplexes

We first investigated if the daunomycin apatamer was able to bind to hemin similarly to the hemin aptamer. It is known that the complexation of hemin to a G-quadruplex structure will lead to a shift in the UV absorption spectra. The complexation of both oligonucleotides to hemin was carried out under UV absorption spectroscopy without the presence of ABTS solution. From the UV-vis absorption spectra (Supplementary Figure 1), Hemin G-Quadruplex and daunomycin G-quadruplex spectra showed a shift with respect to the hemin peak. The peak for hemin is found at 393 nm. Successful complexation of aptamers with hemin will cause the maximum peak to shift to 396 nm. The shift was visible for both aptamers suggesting that both hemin and daunomycin aptamers are able to bind to hemin in a quadruplex. As both quadruplexes contains Na^+^/K^+^ ions, they are able to bind to hemin either by peripheral stacking or intercalation between the G-quadruplexes. Therefore the daunomycin aptamer is able to bind hemin in a quadruplex conformation similar to the hemin aptamer even in absence of the cognate ligand. The characteristic of the daunomycin aptamer is unique as the aptamer was proven to be very robust and independent of the presence of specific ions over a wide pH range. This characteristic is useful as many protein-protein interactions may require varying buffers which may not contain the ions commonly required by the conventional hemin aptamer to form quadruplex structures [10].

### 3.2. Determination of Peroxidase like Activity by Daunomycin Aptamer

The peroxidase activity was measured on the foundation of ABTS^2−^ oxidation in the presence of H_2_O_2_ to produce a coloured radical anion for readout [22,23]. The initial UV spectroscopy analysis of the ligands' (hemin and daunomycin) reactions with ABTS/H_2_O_2_ showed no peaks at 405 nm. The peak at 350 nm corresponds to the background from the non-reacted ABTS development buffer (Supplementary Figure 2). This indicates that both substances independently do not exhibit any catalytic activity or redox activity to promote ABTS reactions. This shows that independently, hemin and daunomycin are not involved in the heterolytic mechanisms of the peroxide.

Two different G-4-containing molecules, the conventional G-4-hemin aptamer as a positive control and daunomycin-specific aptamer were compared. Both G-4 molecules have G-rich stretches with sequence variations. The conditions for complexation were investigated using the control hemin G-4 aptamer to establish the best conditions for G-4 structure formation (Supplementary Figure 2). Similar conditions were used to form the daunomycin G-4 aptamer-hemin complex. The daunomycin G-4 aptamer-hemin complex was able to elicit an oxidative response with ABTS. A peak at 405 nm was observed after the reaction for both aptamers. Our results showed that the signal was slightly lower for daunomycin G-4-aptamer than the control-G-4. The daunomycin G-4-aptamer was able to oxidize the ABTS reaction in the presence of hemin even in the absence of its cognate ligand (Supplementary Figure 2). This shows that the daunomycin aptamer is able to form the G-4 structure to elicit the oxidative reaction independently of daunomycin. Thus in contrast to other quadruplex-forming aptamers, the daunomycin aptamer can function as a highly active DNAzyme independently of its cognate ligand.

### 3.3. Synthesis and Optimization of STV-AuNPs, Bio-DQ and Bio-eGFP as Probe

**Figure 2.**
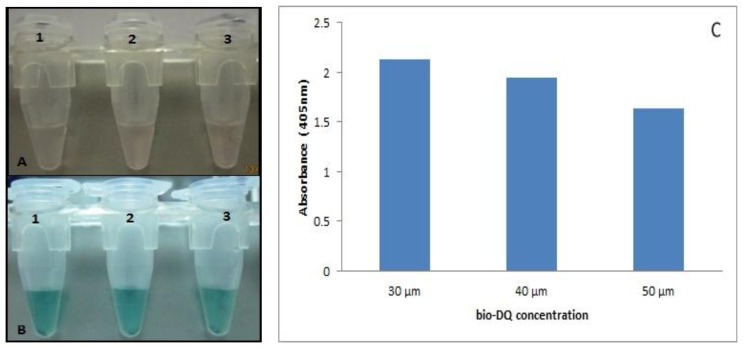
Analysis of antigen-DNAzyme probe based assay. (**A**) pre-ABTS reaction; (**B**) post-ABTS reaction; (**C**) absorbance reading of DNAzyme probes. 200 μg of bio-eGFP was added for each tube with the variations of 30, 40 and 50 μM of bio-DQ added to tubes 1–3, respectively. Two hundred μL ABTS solution added for each reaction and readout was taken at 405 nm. Absorbance readouts were represented in bar chart after 30 min ABTS reaction.

We sought to develop a reporter probe with the daunomycin aptamer to replace the horseradish peroxidase (HRP) commonly used in most immunoassays. We determined the stability of the bio-DQ quadruplex formation on the STV-AuNPs as a colorimetric sensor for the interaction of antigen-antibody binding. The ratio of bio-eGFP to bio-DQ was optimized to provide the best signal readout (Figure 2). The optimal ratio of bio-eGFP to bio-DQ was found to be 1:100 in order to give high colorimetric signal that is 200 μg bio-GFP to 30 μM bio-DQ. 30 μM is the optimum aptamer concentration needed to compete with the antigen with least hindrance due to excess DNA strands. Thus, a decrease in absorbance is witnessed as the amount of DNA increases due to the binding kinetics of both molecules (bio-DQ and bio-GFP) during the mixing process.

### 3.4. Principle of the DNAzyme Probe Immunoassay

In our probe-based immunoassay, the sensitivity is dependent on both the peroxidase activity of the bio-DQ as the signal generator and the affinity of the antigen-antibody physical interaction. For a direct assay, an antibody assay was carried out to show the high affinity binding of the antigen and wells coated with antibody. The absorbance value was used to determine successful capture of proteins by the antibodies. The assay was developed using a scFv that selectively binds the eGFP protein. Negative controls using an anti-ubiquitin scFv and blocking agent controls were analyzed to determine the specificity of the probe to bind the antibody and generate a positive readout. The probe can be used in either a direct or competitive manner for the development of an immunoassay.

**Figure 3.**
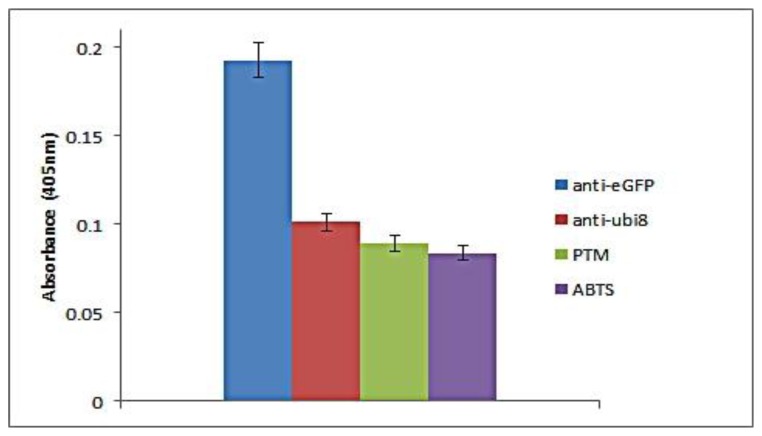
Absorbance analysis of the probe based direct assay for antibody antigen reaction. Reaction time was 30 min with ABTS (1 μg/mL) and H_2_O_2_ (2.2 mM). Bar chart expressed as the average of three independent measurements (*n* = 3).

For the direct assay, the probe was able to bind specifically when the target antigen was present with a clear distinction to the non-specific protein and background (Figure 3). The readout was proportional to the amount of coated antibody. The specificity of the assay is seen when a different antibody (anti-ubiquitin) was coated to the well. The absorbance readout is similar to the background readout. To determine the reproducibility of the direct assay between the probes towards the scFv antibody (anti-eGFP), three different assays were carried out in which 20 μg scFv antibody (anti-eGFP and anti-ubiquitin) and blocking agent were coated on three separate wells in parallel. Based on the bar chart, the assay is reproducible as the antigen-DNAzyme probe strategy could be used for antigen-antibody detection. The current direct assay uses 20 μg of antibody for detection of the probe antigen. The detection limit of the direct assay can be correlated to two aspects, the amount of antibody coated and the amount of antigen present. If sufficient amount of antibody is present but the amount of target antigen is low, this will result in a low readout. The same is expected when high amounts of antigen are present but low amounts of antibodies are available. In addition, the limit of detection for the direct assay would be dependent on the antibody affinities.

**Figure 4.**
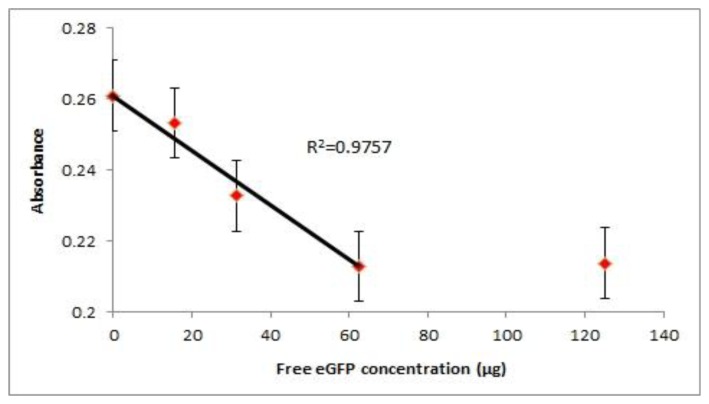
Competitive assay between the probe and the free eGFP against the antibody (scFv format). Linear relationship between the absorbance readout and the concentration of free eGFP (*n* = 3) where each data point represents an average of three absorbance readouts (each error bar indicates the standard errors).

The probe was also applied in a competitive assay where free antigen and the probe antigen compete for the coated antibody. From the experimental data we could see that the limit of detection (LOD) of the probe is around 31.3 μg of free eGFP (Figure 4). In the competitive setup, the signal readout is inversely proportional to the amount of free antigen available. We could see the reduction of signal when 62.5 μg of free eGFP was introduced to compete with the probe for binding while 125 μg of free eGFP was sufficient to reach a plateau. At concentrations lower than 15.7 μg of eGFP, the signal was close to that of no free eGFP concentration. This is due to the maximum antibody binding sites are occupied by the circulating probe. Therefore with the current set up, a maximum amount of competing antigen that is detectable is 62.5 μg. From the graph we can see a linear relationship (R^2^ = 0.9757) between the absorbance readout and concentration of free antigen before it reaches the detection limit. The readout will plateau upon reaching the detection limit hence disrupting the linear correlation of absorbance with the antigen concentration. However, the limit of detection can be improved by introducing less competing probe into the assay. In this manner the dynamic range for signal reduction can be increased for competition assays.

## 4. Conclusions

A simple and rapid direct immuno-based assay was developed using a one pot synthesis of the daunomycin aptamer with the DNAzyme as a reporter system. The use of the biotin and streptavidin interaction allows the antigen-DNAzyme probe system to be applied in a modular form to act as a universal reporter system for immunoassays. The pre-assay generation of the probe eliminates the multiple steps needed in typical ELISA system to introduce secondary antibodies. The design of the probe allows flexibility for both direct and competitive immunoassay applications. The ability of the daunomycin aptamer to form quadruplex structures in a range of conditions even in absence of K^+^ ions is a major advantage for the use in antibody-antigen assays as antibodies and protein-protein interactions may require other buffers. Thus DQ is our choice as it is found to be just slightly less active than HQ. We found that the probe-based system performs better in a direct assay format in comparison to a competitive assay. Nevertheless, we expect that with smaller molecules (haptens) the competitive assay can be more sensitive. In conclusion, the antigen-DNAzyme probe system allows a rapid one-step incubation system as an alternative to conventional immunoassay systems.
