# Supplementary material for: Development of an Antigen-DNAzyme Based Probe for a Direct Antibody-Antigen Assay Using the Intrinsic DNAzyme Activity of a Daunomycin Aptamer
Source: Sensors (Basel). 2013 Dec 27;14(1):346–55. doi: 10.3390/s140100346 (PMC3926560; doi:10.3390/s140100346)
Supplement: Supplementary file 1 [file sensors-14-00346-s001.pdf]

OPEN ACCESS
sensors
ISSN 1424-8220
www.mdpi.com/journal/sensors

Supplementary Information

Development of an Antigen-DNAzyme Based Probe for a Direct Antibody-Antigen Assay Using the Intrinsic DNAzyme Activity of a Daunomycin Aptamer. *Sensors* 2014, *14*, 346-355

Noorsharmimi Omar [1], Qiuting Loh [1], Gee Jun Tye [1], Yee Siew Choong [1], Rahmah Noordin [1], Jörn Glökler [2] and Theam Soon Lim [1,*]

[1] Institute for Research in Molecular Medicine, Universiti Sains Malaysia, 11800 Minden, Penang, Malaysia; E-Mails: mimiez_m5@yahoo.com.my (N.O.); qiutingloh@gmail.com (Q.L.); geejun@usm.my (G.J.T.); yeesiew@usm.my (Y.S.C.); rahmah@usm.my (R.N.)

[2] Department of Molecular Biotechnology and Functional Genomics, Technical University of Applied Sciences Wildau, Bahnhofstr. 1, 15745 Wildau, Germany; E-Mail: gloekler@th-wildau.de

* Author to whom correspondence should be addressed; E-Mail: theamsoon@usm.my; Tel.: +604-653-4801; Fax: +604-653-4803.

Supplementary Figure 1. Normalized UV-Vis spectra analysis of hemin/G-quadruplex structures formation in aqueous solution containing K^+/Na^+ ions carried out in the absence of ABTS reaction with the complex.

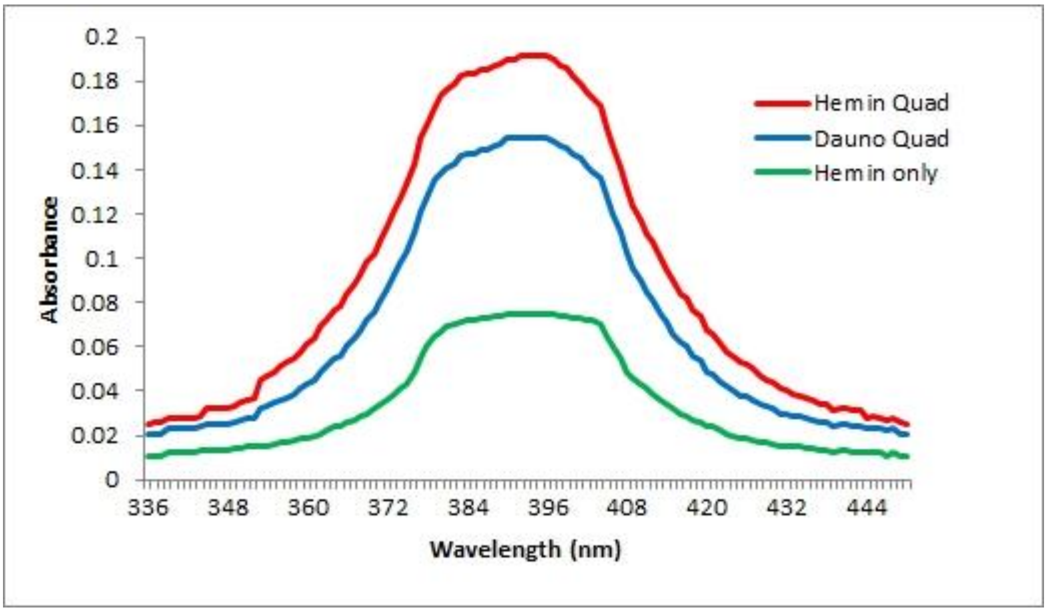

Supplementary Figure 2. UV spectroscopy analysis at 300 nm to 700 nm of peroxide catalyzed oxidation of ABTS. (**A**) 5 mM hemin standard in DMSO and G-4-hemin aptamer with 5 mM hemin in HEPES buffer, pH 7.2 at room temperature; (**B**) 5 mM daunomycin standard in DMSO and daunomycin aptamer in HEPES buffer, pH 7.2 at room temperature. The 405 nm absorbance shows the ABTS reaction with the ligands independently and aptamers.

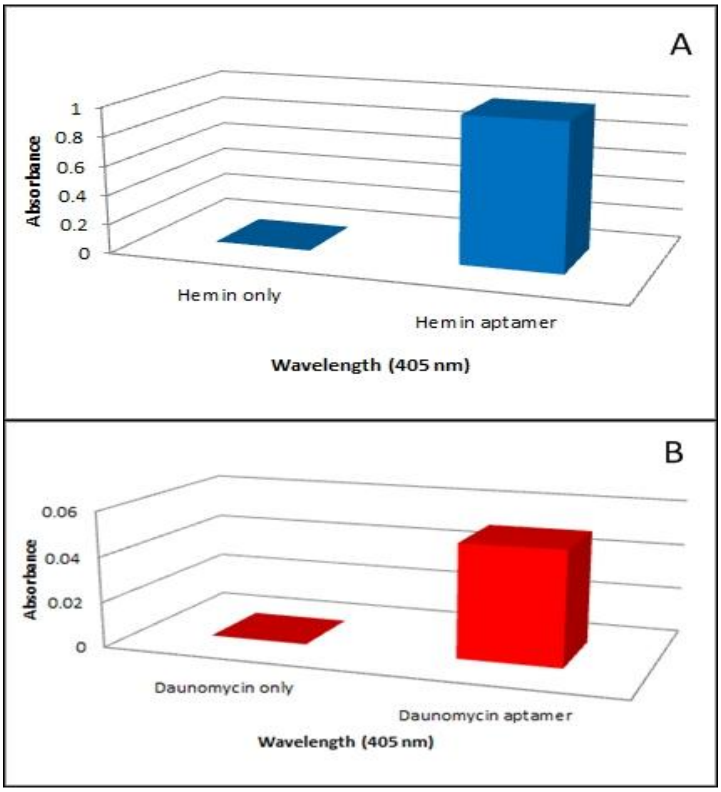

© 2013 by the authors; licensee MDPI, Basel, Switzerland. This article is an open access article distributed under the terms and conditions of the Creative Commons Attribution license (http://creativecommons.org/licenses/by/3.0/).
